# Radioresistance of Stat1 over-expressing tumour cells is associated with suppressed apoptotic response to cytotoxic agents and increased IL6-IL8 signalling

**DOI:** 10.1080/09553000902838566

**Published:** 2009-05-12

**Authors:** Elena V. Efimova, Hua Liang, Sean P. Pitroda, Edwardine Labay, Thomas E. Darga, Vera Levina, Anna Lokshin, Bernard Roizman, Ralph R. Weichselbaum, Nikolai N. Khodarev

**Affiliations:** 1 Department of Radiation and Cellular Oncology, The University of Chicago, Illinois, USA; 2 The Marjorie B. Kovler Viral Oncology Laboratories, The University of Chicago, Illinois, USA; 3 University of Pittsburg Cancer Center, Pennsylvania, USA

**Keywords:** Tumour radioresistance, Stat1, impaired apoptosis, IL6-IL8 signalling, tumour-stromal interactions

## Abstract

**Purpose:**

To determine the mechanisms of Signal Transducer and Activator of Transcription 1 (Stat1)-associated radioresistance developed by nu61 tumour selected in vivo by fractionated irradiation of the parental radiosensitive tumour SCC61.

**Materials and methods:**

Radioresistence of nu61 and SCC61 in vitro was measured by clonogenic assay. Apoptotic response of nu61 and SCC61 cells to genotoxic stress was examined using caspase-based apoptotic assays. Co-cultivation of carboxyfluorescein diacetate, succinimidyl ester (CFDE-SE)-labeled nu61 with un-labeled SCC61 was performed at 1:1 ratio. Production of interleukin-6, interleukin-8 and soluble receptor of interleukin 6 (IL6, IL8 and sIL6R) was measured using Enzyme-Linked Immunosorbent Assay (ELISA).

**Results:**

Radioresistant nu61 was also resistant to interferon-gamma (IFNγ) and the death ligands of tumour necrosis factor alpha receptor (TNFR) family when compared to SCC61. This combined resistance is due to an impaired apoptotic response in nu61. Relative to SCC61, nu61 produced more IL6, IL8 and sIL6R. Using Stat1 knock-downs we demonstrated that IL6 and IL8 production is Stat1-dependent. Treatment with neutralising antibodies to IL6 and IL8, but not to either cytokine alone sensitised nu61 to genotoxic stress induced apoptosis.

**Conclusion:**

Nu61, which over-expresses Stat1 pathway, is deficient in apoptotic response to ionising radiation and cytotoxic ligands. This resistance to apoptosis is associated with Stat1-dependent production of IL6 and IL8 and suppression of 8, 9 and 3.

## Introduction

Tumour cells may acquire radioresistance using multiple pathways including those which are induced/altered by ionising radiation (IR) itself (Lee and Bernstein 1993, Tyrsina et al. 2005, Otero et al. 2006). To investigate IR-induced tumour radio-resistance, a radiosensitive human squamous cell carcinoma tumour, SCC61, was passed and irradiated in vivo. Radioresistant tumours were selected and a tumour cell line, nu61, was isolated (Khodarev et al. 2004). Analysis of the differences in gene expression between cells from differentially selected tumours demonstrated up-regulation of the genes in the Signal Transducer and Activator of Transcription 1 (Stat1) signalling pathway in radioresistant nu61 tumours compared with radiosensitive SCC61 tumours. Recently we and others reported that IR up-regulates Stat1 and a Stat1-dependent pathway in vitro and in vivo (Amundson et al. 2004, Khodarev et al. 2007) and tumour cells resistant to radiation are also resistant to interferon. These results suggested cross–talk between pathways induced by interferons (IFN) and IR. We hypothesised that the development of resistance to the constitutively expressed Stat1 pathway in tumour cells is associated with suppression of the Stat1-dependent apoptotic pathways or/and clonal selection of the cells resistant to Stat1-dependent apoptosis (Khodarev et al. 2007). Next, taking into account that the Stat1 pathway, at least in the context of IFN-signalling, leads to the induction of multiple cytokines, we hypothesised that some Stat1-dependent, pro-survival genes might encode soluble factors secreted by the Stat1 over-expressing cells (Khodarev et al. 2004, Brown et al. 2004, Lehrnbecher et al. 2008). Here we report that indeed apoptotic response in nu61 is impaired resulting in suppression of caspases 3, 7, 8 and 9. This impaired response leads to the resistance not only to IFNs and IR but also to death ligands of tumour necrosis factor alpha receptor (TNFR) superfamily. We also report that nu61 differentially express interleukin-6, interleukin-8 and soluble receptor of interleukin 6 (IL6, IL8 and sIL6R) and this expression is Stat1-dependent. Formation of IL6-IL8-dependent autocrine loops plays a role in nu61 resistance to IR and cytotoxic cytokines. Combined suppression of IL6 and IL8 signalling by neutralising antibodies led to sensitisation of nu61 to genotoxic stress. These data suggest IL6 and IL8 as potential targets for tumour radiosensitisation.

## Materials and methods

### Cell lines

SCC61 and nu61 were cultured in Dulbecco modified Eagle's Media (DMEM-F12) (Invitrogen, CA, USA) supplemented with 20% fetal bovine serum, 2 mM L-glutamine, 100 U/ml penicillin/streptomycin. Cells were maintained at 378C in a humidified chamber with 7% CO2. Stat1 knockdowns and control retroviral vector L4 (Clontech, Palo Alto, CA, USA)-transfected cell lines of SCC61 and nu61 were generated as previously described (Khodarev et al. 2007) and cultured as the parental cell lines. Control vector (CV)-transfected nu61 was named N1L4 (nu61-L4) and CV-transfected SCC61-S1L4 (SCC61-L4). The corresponding cell lines with Stat1 knock-downs were named as NKD and SKD, respectively.

### Reagents

Recombinant human tumour necrosis factor α (TNFα) and propidium iodide (PI) were purchased from Sigma (St Louis, MO, USA). Recombinant human TNF-related apoptosis inducing ligand (TRAIL) was purchased from Peprotech, Inc (Rocky Hill, NJ, USA). Recombinant human interferon γ (IFN-γ), interleukin-6 (IL6) and interleukin-8 (IL8) neutralising antibodies were purchased from R and D Systems (Minneapolis, MN, USA). Anti-Fas (tumour necrosis factor receptor superfamily, member 6; TNFRSF6) CH11 antibody was purchased from Upstate (Charlottesville, VA, USA). Vybrant CFDA-SE (carboxyfluorescein diacetate, succinimi-dylester) cell tracer kit, Vybrant fluoromethyl ketone (FAM) caspase-8 assay kit, Vybrant FAM caspase-3 and -7 assay kits were purchased from Invitrogen (Carlsbad, CA, USA). The caspase-9 detection kit was purchased from Cell Technology, Inc. (Mountain View, CA, USA). The active caspase-3 phycoerythrin (PE) conjugated monoclonal antibody was from BD Pharmigen (San Jose, CA, USA).

### Irradiation

Cells were irradiated using a GE Maxitron Generator operating at 250 kV, 26 mA at a dose rate of 1.18 Gy/min. Samples were collected after irradiation as described in results.

### Clonogenic survival assay

Clonogenic survival assay was performed as described in (Salloum et al. 2000) and analysed as described in (Weichselbaum and Beckett 1987, Hall 1988).

### Flow cytometry

Data were collected on a fluorescence-activated cell sorting FACScan instrument using CellQuest Software (Becton-Dickinson, Franklin Lakes, NJ, USA). At least 10000 events were collected for each sample. FlowJo Software (FlowJo, LLC, Ashland, OR, USA) was used for data analysis. Experiments were repeated 3–4 times per cell line with consistent results.

### Apoptosis/cell death detection assays

Cells were plated in six-well plates at 1 × 10^5^ cells/well in growth medium. After 24 h, cells were left untreated or treated with radiation, IFNγ in designated concentrations (see *Results*), 10 ng/ml TNFα, TRAIL, or Fas-activating antibody CH11. 24 or 48 h later cells were harvested, washed in cold phosphate buffer saline (PBS) and stained for 5 min in 1 μg/ml propidium iodide (Sigma, St Louis, MO, USA), followed by FACS analysis, to detect late apoptotic or necrotic cells. Cells with active caspase-3 were detected by analysing the fluorescein isothiocyanate (FITC)-positive population using Vybrant FAM caspase-3 kit (Invitrogen) according to the manufacturer's protocol. For measurement of activation of caspases 8, 9 and 3/7 in cell cultures we used luminescent-based Caspase-Glo® assays, provided by Promega (Madison, WI, USA).

### Co-cultivation of nu61 and SCC61

In experiments where the cells are co-cultured in physical contact with each other, nu61 were labeled with CFDA-SE and placed in co-culture with unlabeled SCC61 in a 1:1 ratio. Treatment with 3 Gy IR, 50 ng/ml IFNγ and 10 ng/ml TNFα was begun 24 h later and cells harvested for flow cytometry 48 h later.

### Measurement of IL6, IL8 and sIL6R secretion in CV-transfected and Stat1 knock-downed SCC61 and nu61 cell lines

S1L4, N1L4, SKD and NKD were plated at concentrations of 0.3–0.5 × 10^6^ cells/well in six-well plates with 2.0 ml of growth media. Some 18–24 h later cells were irradiated, incubated for an additional 48 h and samples of conditioned media were collected for ELISA analysis. In preliminary experiments, we compared irradiation at 3 and 6 Gy and found that in these conditions 3 Gy led to a more pronounced induction of IL6, IL8 and sIL6R (Khodarev et al. 2001). Therefore this dose (3 Gy) was used in all subsequent experiments. Each cell line was plated in three independent wells. ELISA kits were obtained from R & D Systems (Minneapolis, MN, USA) and concentrations of IL6, IL8 and sIL6R were determined according to manufacturer's instructions.

### Neutralisating antibodies against IL6 and IL8

To determine the effect of neutralising antibodies (N-Ab) against IL6 and IL8 on cell survival and IR-resistance, 30,000 nu61 cells/well were plated on 96-well plates. 24 hours later cells were treated with N-Ab against IL-6 (1 μg/ml, R & D Systems), IL8 (20 μg/ml, R & D Systems), or both, 4 h before treatment with IR or IFNγ (50 ng/ml). 24–48 h after treatment, caspase 3 and 7 activation were determined using Caspase-Glo® assay (Promega) and by FACS analysis after cell staining with an antibody specific for the active form of caspase-3.

### Statistical analysis

All experiments were performed at least three times. Quantitative data are presented as mean ± SE. Significance of difference was estimated by Student's two-tailed *t*-test with cut-off *p* ≤ 0.05.

## Results

### Nu61 are more radioresistant in vitro compared to SCC61 based on clonogenic survival assay

**Figure 1:**
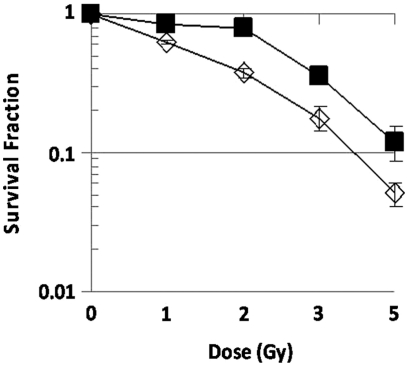
Clonogenic survival of nu61 (black squares) and SCC61 (white diamonds). Cells were plated at P60 (three dishes per dose) and 18 hours after plating irradiated as indicated in *Methods*. Data were analysed as described in *Methods* (see also *Results* for details). Experiments were repeated three times. Shown are mean values; error bars-standard error (SE).

In our previous reports we demonstrated that the nu61 tumour, selected from the SCC61 tumour by in vivo fractionated irradiation, is more radioresistant based on in vivo assays and overexpresses Stat1 (Khodarev et al. 2004, 2007). In the current experiments we directly compared in vitro clonogenic survival of SCC61 and nu61. It has been shown previously that the parental SCC61 has very low clonogenic ability (Quiet et al. 1991). We therefore used a relatively low range of doses (between 0 and 5 Gy). As is shown in Figure 1, the major difference between the two cell lines was observed between 0 and 2 Gy as a pronounced shoulder in nu61. We used a biphasic model described in (Hall 1988) and previously used by us for correlation of tumour radioresistance in vitro and in vivo (Weichselbaum and Beckett 1987). We found that between 2 and 5 Gy, D_0_ values for SCC61 and nu61 were 0.66+/−0.03 and 0.60+/−0.07, respectively (mean ± SE, *p* > 0.05). Extrapolation number (*n*) was higher for nu61 compared to SCC61 (3.46 ± 2.36 and 1.43 ± 0.14 respectively; mean ± SE) but these differences were also not significant (*p* > 0.05). However, estimation of D_1_ in the dose range between 0 and 2 Gy revealed a significant difference between nu61 and SCC61 (2.75 ± 0.03 and 0.99 ± 0.03; *p* < 0.05). The larger D_1_ value in nu61 may be attributed to increased sublethal damage repair (Weichselbaum and Beckett 1987, Wang et al. 2008). Literature indicates that sublethal damage repair is connected with increased resistance to genotoxic stress associated with suppressed apoptosis (Blenn et al. 2006, Hara et al. 2008).

### Nu61 demonstrates impaired apoptotic response to ionising radiation and interferons

**Figure 2:**
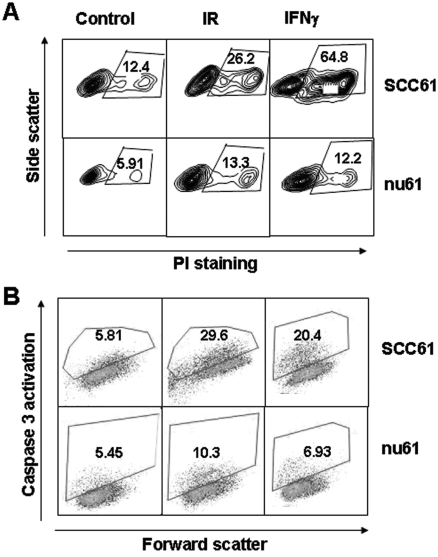
Down-regulation of caspase-3 activation in nu61 results in decreased cell death in response to treatment with IR and IFNγ. 48 hours after a single dose of 6 Gy IR, or 50 ng/ml of IFNγ nu61 and SCC61 cells were either stained with PI (panel A) as a measure of cell death or active caspase-3 (panel B) to measure apoptosis. Nu61 cells demonstrate both reduced total cell death (panel A) and apoptosis (panel B) compared to SCC61 in response to IR and IFNγ (see text for details). Shown are representative data of three independent experiments.

To test the hypothesis that the suppression of cell death in nu61 following IR and IFNγ is due to the suppression of the apoptotic response located downstream from Stat1, we analysed cell death and the apoptotic response in nu61 and SCC61 cell lines. We used PI staining at 48 h as an index of total cell death, and measured apoptosis by flow cytometry for detection of cells that express the proximal caspase-3 as described in *Methods*. All experiments were repeated at least three times, and representative data are shown. As shown in Figure 2A, ionising radiation (6 Gy) induced 26.2% (23.3 ± 8.3%) PI positive cells in SCC61 and 13.3% (10.33 ± 3.4%) PI positive cells in nu61. Figure 2B shows the same trend for the post-IR accumulation of caspase-3-positive cells in SCC61 and nu61 (see *Methods*). 29.6% (27.5 ± 2.9%) of caspase-3-positive cells accumulated in SCC61 48 hours post-IR and only 10.3% (9.8 ± 1.5%) in nu61 (*p* = 0.005). These data show that the differences in post-irradiation survival between nu61 and SCC61 are mediated by caspase-3 mediated apoptosis which is suppressed in nu61.

Figure 2 shows also the response of SCC61 and nu61 to IFNγ (50 ng/ml). Forty-eight hours following 50 ng/ml IFNγ, 64.8% (61 ± 5.8%) of PI positive cells were detected in SCC61 and only 12.2% (13.8 ± 1.3%; *p* < 0.0001) in nu61 (Figure 2A). Accumulation of caspase-3-positive cells was simultaneous with PI-positive cells (Figure 2B). 48 hours post IFNγ treatment 20.4% (19.8 ± 2.1%) of caspase-3-positive cells were detected in SCC61 and only 6.93% (7.3 ± 1.5%) in nu61 (*p* = 0.03). Pretreatment of both cell lines with the caspase-inhibitor Z-Val-Ala-Asp (OCH_3_)-fluoromethylketone (Z-VAD-FMK) abolished apoptotic response to both IR and IFNγ (data not shown). We concluded from these experiments that interferon and radiation-induced cell death can be accounted for by activation of caspase-3-mediated apoptosis, and the decrease in the apoptotic response, at least in part, accounts for the suppressed sensitivity to IFN and IR in nu61 relative to SCC61.

### Resistance to IR and interferons correlates with the resistance to death ligands

**Figure 3:**
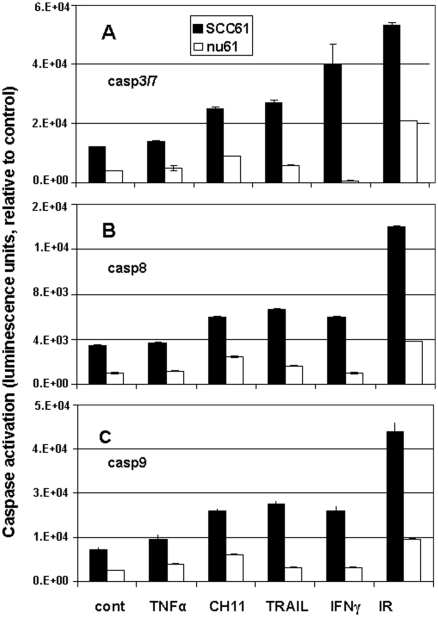
Proximal and effector caspases activation is suppressed in nu61 compared to SCC61 in response to genotoxic stress and death ligands of the TNF superfamily. Activation of the proximal caspases of the extrinsic (panel B) and intrinsic (panel C) apoptotic pathways, as well as the effector caspases-3 and -7 (panel A) common to both pathways were measured after treatment with 10 ng/ml TNFα, 25 ng/ml CH11 (Fas-activating antibody), 100 ng/ml TRAIL, 6 Gy IR or 50 ng/ml IFNγ at 24 h after treatment for caspases-8 and -9 and 48 h for caspases-3 and -7. For all measurements we used Caspase-Glo® kits (see *Methods*). Black bars represent SCC61, white bars represent nu61. Error bars represent standard deviations (*n* = 3). *p* values are less than 0.05 for all graphs, representing significant differences between SCC61 and nu61.

We next tested the hypothesis that resistance to genotoxic stress and interferons correlates with the resistance to death ligands of TNFR superfamily. We studied the cytotoxic effects of TNFα, CH11 (FAS-activating antibody) and TRAIL (TNF-related apoptosis inducing ligand) in nu61 and SCC61 cells. As is shown in Figure 3, each ligand, as well as irradiation and IFNγ, led to significant apoptotic death in SCC61 compared to nu61 (significance was estimated by *t*-test with cut-off value of *p* < 0.05). Both proximal caspases (CASP8 and CASP9) were activated in SCC61 compared with nu61, indicating that both intrinsic and extrinsic pathways of apoptosis (Kim 2005, Fulda and Debatin 2006) are functional in the parental SCC61 cell line, but are suppressed in nu61. Effector caspases 3/7 were also suppressed in nu61 compared to SCC61 confirming that the cell death detected in our experiments is connected with apoptosis. Similar data were detected in flow cytometry experiments (data not shown). These data show that overall cell death induced by the TNFR family ligands, IFNγ and IR was mediated by apoptosis in both cell lines and the resistance of nu61 to IR, IFNs and the TNFR family ligands is due to the suppression of apoptotic caspases 3, 8 and 9.

### Co-cultivation of nu61 with SCC61 partially protects SCC61 from apoptosis

**Figure 4:**
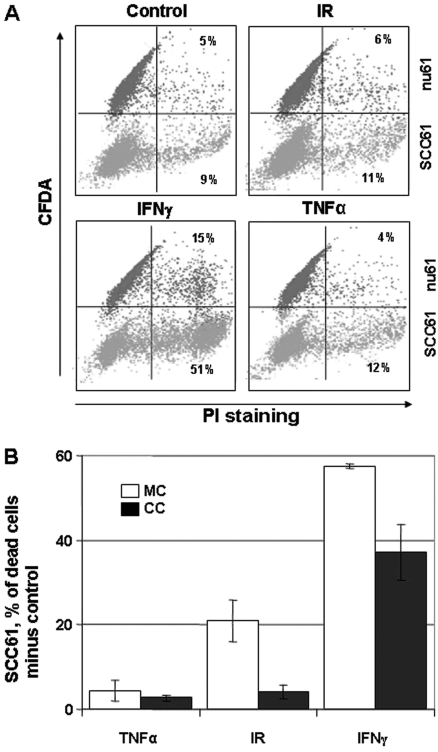
Interaction of nu61 and SCC61 cells partially protects SCC61 from cell death and is associated with differential secretion of IL6, sIL6R and IL8. Nu61 cells were labeled with CFDA-SE (upper quadrants in panel A) to distinguish them from SCC61 (lower quadrants of panel A). Cells were co-cultured and treated as described in *Methods*. Although nu61 do not completely rescue SCC61 from cytotoxic treatments (panel A), SCC61 cells in co-culture compared with SCC61 cells in monoculture (panel B) are more resistant to cell death. Shown on panel B are mean values of three independent experiments +/− SE.

In the next set of experiments, we co-cultivated SCC61 with nu61 to see if SCC61 could be rescued by the presence of proteins secreted by nu61. Nu61 cells were labeled with CFDA (see *Methods*) mixed with unlabeled SCC61 at a 1:1 ratio, and 24 h later, treated with IR (3 Gy), IFNγ (50 ng/ml) or TNFα (10 ng/ml). 48 h after treatment, cell death was determined by PI staining using flow cytometry. Control experiments were run in which monocultures of the same cell lines were subjected to the same treatments. Figure 4A shows that in the co-culture with nu61 SCC61 is more sensitive to cytotoxic treatments than nu61, consistent with our previous data. However, comparing the relative amount of cell death of SCC61 in monoculture with SCC61 in co-culture, we found that co-culture protects SCC61 from cytotoxic stimuli. Figure 4B show that treatment by TNFα did not lead to significant difference between SCC61 cultivated in mono-culture or co-culture with nu61. However, treatment by IR led to the significant 5.5-fold protection (*p* = 0.023) and treatment by 50 ng/ml of IFNγ led to 1.6-fold protection of SCC61 in co-culture relative to monoculture (*p* = 0.026: see Figure 4B). These data suggested that nu61 can produce some secreted factors, which provide protection of the parental SCC61 cells from the cytotoxic insult.

### Identification of the pro-survival factors secreted by nu61

**Figure 5:**
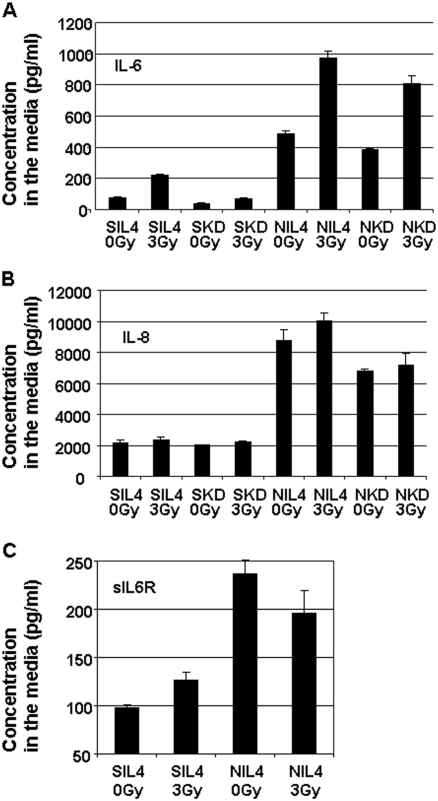
Production of IL6 (panel A), IL8 (panel B) and sIL6R (panel C) by CV-transfected nu61 and SCC61 and cells with stable Stat1 knock-down. Cells were plated in six-well plates and 24 hours later irradiated by 3 Gy as described in *Methods*. Each cell line was plated in three independent wells. 48 hours conditioned media was collected and used for estimation of concentrations of IIL6, IL8 and sIL6R using ELISA kits (R & D Systems, Minneapolis, MN, USA). See text for explanations. Error bars=SE.

To identify pro-growth/pro-survival factors produced by nu61, we performed pilot experiments using Luminex technology (Levina et al. 2008). Out of 35 cytokines tested we found that, IL8 and soluble IL6 receptor (sIL6R) were differentially produced by nu61 relative to SCC61 (data not shown). To investigate the relative expression of IL6, IL8 and sIL6R in nu61 and SCC61 we used ELISA (see *Methods*). Since we were interested in the potential relationships between Stat1 expression and the expression of IL6, IL8 and sIL6R, we used stable Stat1 knock-downs and CV-transfected cell lines of SCC61 and nu61. We showed previously that stable Stat1 knock-down by retroviral-based shRNA led to 2.5-fold suppression of Stat1 protein in NKD relative to N1L4 (Khodarev et al. 2007). Furthermore we demonstrated that this knock-down leads to significant 1.9-fold radiosensitisation of N1L4 in vivo. These data indicated that some prosurvival/radioprotective pathways may operate down-stream from Stat1 (Khodarev et al. 2007). We therefore suggested that IL6-IL8 signalling may participate in these pathways. First we compared production of IL6, IL8 and sIL6R by N1L4 relative to S1L4 (control cell lines, see *Methods*). Figure 5A–C shows that all three ligands were expressed in N1L4 at the higher levels compared to S1L4. For IL6 at the basal level the differences between S1L4 and N1L4 were highly significant (6.9-fold increase in N1L4; *p* < 0.00001). For IL8 there was a 4.1-fold increase with significance *p* = 0.0007. For sIL6R N1L4 media was enriched by this ligand 2.4-fold compared to S1L4 with *p* = 0.00057. Taken together, these data show that the nu61 phenotype is associated with increased production of IL6, IL8 and sIL6R compared to the parental SCC61 cell line.

Next we compared differences in the production of IL6, IL8 and sIL6R in control cell lines (S1L4 and N1L4) and the corresponding Stat1 knock-downs (SKD and NKD, see *Methods*). Basal IL-6 production in SKD was decreased compared to the S1L4 1.97-fold (*p* = 0.0004; see Figure 5A). For NKD/N1L4 suppression of IL6 production was 1.3-fold with *p* = 0.01 (see Figure 5A). This suggests that IL-6 production is Stat1-dependent in both cell lines. For basal IL-8 production we did not detect any trends in SCC61, perhaps due to the low sub-threshold expression of this cytokine, but in nu61, we detected significant suppression of IL-8 production in NKD relative to N1L4 (see Figure 5B). NIL4 produced 8785.7 ± 695.8 pg/ml of IL-8, while NKD produced only 6811.6 ± 114 pg/ml. The difference was significant with *p* = 0.046. These data suggest that at least in N1L4 IL8 production is also associated with Stat1 expression, as is IL6 production (see above).

Finally we examined the effects of IR on the expression of IL6, IL8 and sIL6R. As is shown in Figure 5A, IL6 demonstrates clear up-regulation post IR both in SIL4 and N1L4. For S1L4 on the basal level the concentration of IL6 was equal to 78.1 + 2.92 pg/ml. 48 hours post-IR concentration increased to 220.1 ± 8.28 pg/ml. This indicates a 2.8-fold induction with significance *p* = 7.95E-05. In N1L4 the basal concentration of IL6 was equal to 488.1 ± 20.1 pg/ml. 48 hours post IR the concentration increased to 972.1 ± 42.3 pg/ml (1.99-fold induction; *p* = 0.00046). These data demonstrate that IL6 production is induced by IR in both SIL4 and N1L4 cell lines. Interestingly, together with IR-induced up-regulation of IL6 in S1L4, we also detected IR-induced up-regulation of sIL6R (see Figure 5C). The basal concentration of sIL6R in S1L4 was 97.5+/−2.93 pg/ml. 48 hours post IR it increased to 126.5+/−8.4 pg/ml. The fold-induction was relatively modest (1.3-fold) but statistically significant (*p* = 0.0297). Contrary to these observations, in N1L4 irradiation shows a trend towards the suppression of sIL6R production (0.82-fold), but without significance (*p* = 0.1932). In other words, in S1L4, IR led to the up-regulation of both IL6 and sIL6R, while in N1L4, IR significantly induced only IL6 production, but not sIL6R. These data are consistent with recent observations that shedding of IL6 receptor is connected with activation of apoptosis (Chalaris et al. 2007). Sensitivity of SCC61 to apoptosis (see Figures 2 and 3) is also consistent with these data.

The data described in Figure 5 led to three important conclusions. First, N1L4 overexpress IL6, IL8 and sIL6R on the basal level compared to the parental cell line S1L4. Pro-survival and anti-apoptotic signalling connected with these cytokines may at least in part explain the resistance of nu61 to apoptosis compared to SCC61. Second, comparing wild type (wt) and Stat1 KD variants of both cell lines we found that both IL6 and IL8 production are dependent on Stat1. To our knowledge this is the first observation that in SCC61/nu61 cell lines Stat1 expression is associated with production of IL6 and IL8. Third, we found that IL6/sIL6R system is clearly radioinducible in SCC61 (see *Discussion*).

### Inactivation of IL6 and IL8 sensitises cells to genotoxic stress and IFNγ in nu61

**Figure 6:**
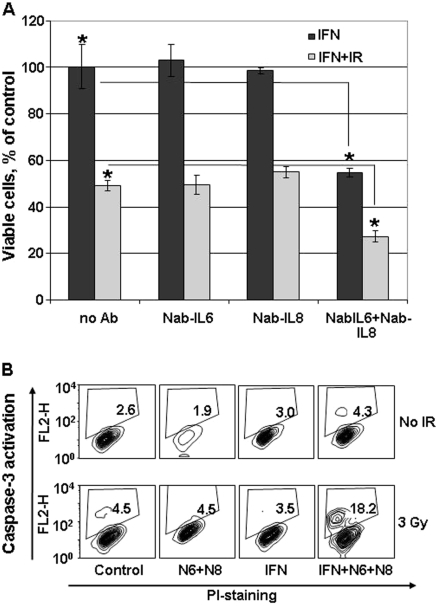
Cell viability of nu61 cells is reduced by neutralising antibodies to IL-6 and IL-8 and is associated with activation of apoptotic cell death. Cells were treated with either IFNγ (50 ng/ml) alone or in combination with IR (6 Gy) (see *Methods*). The antibodies to IL-6 and IL-8 together, but not alone, reduced viability of nu61 in response to both IFNγ alone and in combination with IR, with significance at *p* < 0.001 (panel A). Data on panel A are normalised to the cells treated by 50 ng/ml of IFNγ, but not treated by neutralising Abs (indicated as no Ab on X-axis). Staining for active caspase-3 (panel B) shows that neutralising antibodies to IL-6 and IL-8 activate caspase-3 in response to IFNγ + IR (representative data from three experiments). Black bars on the panel 5A represent nu61 cells treated by IFNγ and combinations of neutralising Abs to IL6 and IL8. Grey bars represent the same cells treated by the combination of IR and IFNγ. Error bars are standard deviations (*n* = 3) and asterisks indicate pairs with *p* values < 0.05.

To define the role of IL6 and IL8 in nu61 survival we used neutralising antibodies to IL-8 and to IL-6 alone and in combination. Cells were treated with neutralising antibodies (N-Ab) as described in the *Methods*. As shown in Figure 6A, neither antibody alone affected survival of nu61 after treatment with IFNγ alone or in combination with IR (see *Methods*). However, the combination of both antibodies led to a 2-fold suppression of nu61 viability after treatment with IFNγ and a 1.9-fold suppression after combined treatment with IR and IFNγ. The significance of these observations was confirmed by t-test with *p* < 0.001 (Figure 6A). We investigated the action of neutralising antibodies to IL-6 and IL-8 on the activation of apoptotic caspases-3 in response to IR and IFNγ. As is shown in Figure 6B, neutralisation of both IL6 and IL8 led to a 4-fold increase of caspase-3-positive cells treated by the combination of IR (3 Gy) and IFNγ (50 ng/ml).

Our data indicate that treatment of parental SCC61 with recombinant IL6 and IL8 led to partial protection from IR-induced apoptosis (3 Gy) by 31.5% (not shown). Further experiments with stably transfected cell lines are necessary to fully understand the mechanisms of this protection.

## Discussion

In previous reports we described the selection of a human radioresistant, IFN-resistant tumour cell line, nu61, by serial passage and fractionated irradiation in nude mice. We showed that radioresistance of nu61 is associated with the constitutive up-regulation of the Stat1 pathway and Stat1-dependent, IFN-stimulated genes (ISG) (Khodarev et al. 2004, 2007). We also showed that IR can directly activate the Stat1 pathway and that selection of parental interferon/radiosensitive tumours-SCC61 against in-terferons leads to the selection of clones that are cross-resistant to IR and IFN and over-express genes in the Stat1 pathway. At the same time Stat1 knockdown leads to radiosensitisation of nu61 (Khodarev et al. 2007). These observations suggested that constitutive over-expression of the Stat1 pathway leads to combined resistance to IFN and IR, and is associated with Stat1-dependent pro-survival signalling. However, the mechanisms of this cross-resistance and pro-survival signalling remained elusive.

Cytotoxicity following Stat1 activation by interferon stimulation is mediated by apoptosis (Samuel 1991). We therefore hypothesised that nu61 resistance to genotoxic stress is mediated, at least in part by resistance to apoptosis induction. Activation of the apoptosis-associated caspases is the most common hallmark of apoptotic cell death (Eckelman et al. 2006, Youle and Strasser 2008). Using various approaches for the measurement of caspases 3, 7, 8 and 9 we found that they were suppressed in nu61 relative to the SCC61. These data indicate impaired apoptotic response in nu61. This indicates that nu61 resistance to genotoxic stress is located down-stream from Stat1, contrary to the few reported cases of up-stream resistance to IFN (Kaplan et al. 1998, Muller et al. 2005). Interestingly, according to clonogenic assay, in the region of the initial slope (see Figure 1) the number of clonogenic cells in nu61 exceeds that in SCC61 by approximately two-fold. This is the inverse of the proportion of caspase-3 positive apoptotic cells in the short-term assay (see Figure 2). This suggests that decreased apoptosis in nu61 may be connected with increased proportion of apoptosis-resistant clonogenic cells in nu61 population, which is consistent with our results about increased production of growth/proliferation-stimulating cytokines in nu61 (see below).

We further suggested that suppression of apoptotic cell death in nu61 might lead to the increased resistance of this cell line and corresponding tumours to the death ligands of the TNFR superfamily, which mostly operate through induction of apoptosis (Varfolomeev et al. 2005, Dai et al. 2006, Baritaki et al. 2007). Indeed, our results show that in addition to IR and IFN resistance, nu61 relative to SCC61 is also resistant to TNFα, TRAIL and FAS. All of these ligands are known to be involved in the control of tumour growth (Weichselbaum et al. 2002, Han et al. 2008). It is possible that the selection of radioresistant tumour clones induced by fractionated irradiation is associated with suppression of apoptotic pathways activated by different pro-inflammatory cytokines (Dunn et al. 2005).

Our previous data also indicated that in nu61 the functions of Stat1 are connected with pro-survival and radio/chemo-protective signalling (Khodarev et al. 2007, Weichselbaum et al. 2008). We should note that these observations are contradictory to traditional understanding of Stat1 as tumour suppressor gene (Samuel 2001, Levy and Darnell 2002). However, currently several laboratories have confirmed the association of Stat1 with an aggressive chemo-/radio-resistant and oncogenic phenotype.

It has been shown that Stat1 can control essential pro-survival genes, such as Myeloid Cell Leukemia 1 (MCL-1) (Timofeeva et al. 2006), Interferon-Induci-ble Transmembrane Protein 1 (IFITM1) (Kita et al. 2003) and multi-drug resistance Major Vault Protein MVP (Steiner et al. 2006). Constitutive over-expression of Stat1 and Stat1-dependent genes is associated with protection of tumour cells from genotoxic stress following treatment with fludarabine (Friedberg et al. 2004), doxorubicin (Thomas et al. 2004), cis-platinum (Roberts et al. 2005) and the combination of ionising radiation and doxorubicin (Rickardson et al. 2005, Fryknas et al. 2007). Most recently it was demonstrated that suppression of Stat1 leads to radiosensitisation in renal cell carcinoma (Hui et al. 2009), which is consistent with our previous observations (Khodarev et al. 2007). In the current report we provide a partial mechanistic explanation for such a ‘reversed’ Stat1-dependent phenotype that is connected with alterations in apoptosis. Another part of an explanation may be connected with secretion of Stat1-dependent pro-survival factors (Adams and Cory 2002, Tan and Coussens 2007). To test this hypothesis we established co-cultures of nu61 and SCC61 (see Figure 4) and found that nu61 secretes factors which partially rescue SCC61 from cell death. We identified these factors as IL8, IL6 and the soluble receptor of IL6 (sIL6R), which were differentially secreted by nu61 compared to SCC61 (see Figure 5). IL6 and IL8 signalling are recognised as important factors of tumourogenesis (Bachelor and Bowden 2004, Hodge et al. 2005, Nicolini et al. 2006) and may be associated with the Stat1 pathway through glycoprotein 130 (gp130), CCAAT/enhancer binding protein beta (C/EBP beta) and direct interaction of Stat1 with IL8 promoter (Ernst and Jenkins 2004, Yamaoka et al. 2004, Hu and Nicholas 2006, Galdiero et al. 2006). Our experiments show that expression of IL6 and IL8 is directly suppressed by Stat1 knock-down (see Figure 5). These experiments suggest that expression of these cytokines is controlled by Stat1, but additional experiments are needed to decode these relationships.

Of specific interest are our observations about sIL6R. This receptor does not individually activate down-stream signalling. Upon binding with IL6 it further binds to the ubiquitous receptor gp130, and activates Stat1/Stat3 pathways and pro-survival Ras and Akt pathways (Ernst and Jenkins 2004). These processes called ‘trans-signalling’ and solubilisation (shedding) of sIL6R are associated with activation of apoptosis (Chalaris et al. 2007). These data are consistent with our observations presented in Figure 4 and Figure 5 and suggest that nu61 cells may not only promote their own growth and survival but also form pro-survival paracrine loops, at least in part involving IL8/IL6/sIL6R network.

Our experiments show that this phenotype can be reversed with neutralising antibodies to IL6 and IL8 (see Figure 6). These results provide compelling evidence that IL6-IL8 signalling is associated with resistant phenotype of nu61.

Based on our results we hypothesise that Stat1, IL6/sIL6Rα and IL8 form an interdependent network associated with increased survival of nu61. The parental tumour clone (SCC61) is subjected to negative pressure from irradiation and/or the tumour microenvironment which produce multiple factors able to activate the Stat1 pathway (Nishigaki et al. 2006, Buess et al. 2007, Andersen et al. 2008). Importantly, our data suggest that IR-induced up-regulation of IL6 in parental SCC61 tumour cells might be one of the initial events in the activation of the Stat1 pathway (see Figure 5). This activation leads to the elimination of the majority of the tumour cells, sensitive to Stat1-delivered cytotoxicity but also induces the selection of ‘nu61-like’ clones, which are resistant to irradiation and the stromal death ligands due to suppression of Stat1-dependent apoptotic pathways. The ability of these cells to secrete pro-survival ligands promotes growth and the transformation of surrounding less aggressive tumour or pre-malignant clones. This selection leads to the formation of tumour clones with aggressive properties and combined resistance to IR, chemotherapy and cytotoxic cytokines.

Our previous and current data suggest that targeting Stat1, IL6 and IL8 may enhance the therapeutic ratio for treatment of Stat1 over-expressing tumours.
