# Erratum

**DOI:** 10.1080/09553000903094797

**Published:** 2009-06-24

Radioresistance of Stat1 over-expressing tumour cells is associated with suppressed apoptotic response to cytotoxic agents and increased IL6-IL8 signalling. *International Journal of Radiation Biology* 2009;85:421–431.

*International Journal of Radiation Biology* hereby notifies its readers that one word was erroneously omitted from the Abstract. The article published online with this error and consequently its PubMed record must be considered incorrect. The error is highlighted in bold type below.

*Conclusion:* Nu61, which over-expresses Stat1 pathway, is deficient in apoptotic response to ionising radiation and cytotoxic ligands. This resistance to apoptosis is associated with Stat1-dependent production of IL6 and IL8 and suppression of **caspases** 8, 9 and 3.

